# Sporadic Inclusion Body Myositis at the Crossroads between Muscle Degeneration, Inflammation, and Aging

**DOI:** 10.3390/ijms25052742

**Published:** 2024-02-27

**Authors:** Valeria Guglielmi, Marta Cheli, Paola Tonin, Gaetano Vattemi

**Affiliations:** 1 Cellular and Molecular Biology of Cancer Program, NCI-Designated Cancer Center, Sanford Burnham Prebys Medical Discovery Institute, La Jolla, CA 92037, USA; vguglielmi@sbpdiscovery.org; 2 Immunity and Pathogenesis Program, Infectious and Inflammatory Disease Center, Sanford Burnham Prebys Medical Discovery Institute, La Jolla, CA 92037, USA; 3 Department of Neurosciences, Biomedicine and Movement Sciences, University of Verona, 37134 Verona, Italy; marta.cheli@univr.it (M.C.); paola.tonin@univr.it (P.T.)

**Keywords:** sporadic inclusion body myositis (sIBM), muscle fiber degeneration, protein aggregation, inflammation, aging, inflammaging

## Abstract

Sporadic inclusion body myositis (sIBM) is the most common muscle disease of older people and is clinically characterized by slowly progressive asymmetrical muscle weakness, predominantly affecting the quadriceps, deep finger flexors, and foot extensors. At present, there are no enduring treatments for this relentless disease that eventually leads to severe disability and wheelchair dependency. Although sIBM is considered a rare muscle disorder, its prevalence is certainly higher as the disease is often undiagnosed or misdiagnosed. The histopathological phenotype of sIBM muscle biopsy includes muscle fiber degeneration and endomysial lymphocytic infiltrates that mainly consist of cytotoxic CD8^+^ T cells surrounding nonnecrotic muscle fibers expressing MHCI. Muscle fiber degeneration is characterized by vacuolization and the accumulation of congophilic misfolded multi-protein aggregates, mainly in their non-vacuolated cytoplasm. Many players have been identified in sIBM pathogenesis, including environmental factors, autoimmunity, abnormalities of protein transcription and processing, the accumulation of several toxic proteins, the impairment of autophagy and the ubiquitin–proteasome system, oxidative and nitrative stress, endoplasmic reticulum stress, myonuclear degeneration, and mitochondrial dysfunction. Aging has also been proposed as a contributor to the disease. However, the interplay between these processes and the primary event that leads to the coexistence of autoimmune and degenerative changes is still under debate. Here, we outline our current understanding of disease pathogenesis, focusing on degenerative mechanisms, and discuss the possible involvement of aging.

## 1. Introduction

Sporadic inclusion body myositis (sIBM) is a chronic progressive muscle disease that primarily affects people 50 years and older [1,2]. It is the most common acquired myopathy in the elderly, with a prevalence ranging between 5 and 180 per million, depending on the geographical area [3,4,5,6]. sIBM is more common in males than females (2:1) and is associated with higher morbidity and mortality [5,6]. The disease presents with muscle weakness mainly affecting the quadriceps and finger flexors [6]. At present, there are no disease-modifying therapies for this progressive disease that eventually leads to severe disability [7]. Despite common clinical characteristics, the phenotype can be variable, and the diagnosis relies on the combination of clinical evaluation, laboratory tests, and pathologic changes in muscle biopsy [6]. Histological hallmarks include both degenerative features such as protein aggregation in myofibers and autoimmune aspects such as endomysial infiltration by T cells [8,9].

sIBM is classified among the idiopathic inflammatory myopathies (IIM) along with dermatomyositis (DM), polymyositis (PM), and immune-mediated necrotizing myopathy (IMNM), but the lack of response to immunosuppressive treatment by sIBM patients have raised questions about the relevance of immune processes in disease pathogenesis [8,10,11].

Even though several studies have uncovered the processes participating in the degenerative and immune responses occurring in the disease, the relationship between these two aspects still remains unknown. Improving our knowledge of the pathogenic mechanisms is necessary to better understand this disorder, identify therapeutic targets, and design effective therapies for patients. Here, we provide an overview of the clinic, histopathology, and disease mechanisms of sIBM and discuss the contribution of skeletal muscle and immune system aging to the disease.

## 2. Diagnosis of sIBM

### 2.1. Clinical Aspects

sIBM is clinically characterized by slowly progressive asymmetrical muscle weakness, predominantly affecting the quadriceps, deep finger flexors, and foot extensors [1,8]. Pharyngeal muscles often become impaired, resulting in dysphagia [12]. Head drop and camptocormia may occur, and facial muscles are occasionally affected [13,14]. Late stages of the disease are characterized by weakness and atrophy of distal and proximal muscles and possible impairment of neck flexors and extensors [15]. Sensory function is normal, and deep tendon reflexes are preserved, but when atrophy of major muscles occurs, the latter can be abolished [16]. Disease progression is usually slow. Variability in the clinical presentation and a lack of specific features/muscle involvement in some patients represent challenges for a prompt and correct diagnosis, making laboratory tests and histological analysis of muscle biopsy fundamental tools in the diagnostic workup [7].

### 2.2. Laboratory Studies

CK levels are frequently normal or only mildly elevated, usually less than 10 times the upper normal values [9,17].

Autoantibodies are considered a useful tool in the diagnosis of IIM. Based on their specificity, autoantibodies in IIM are traditionally classified as myositis-specific (MSA) or myositis-associated antibodies (MAA), with the latter also occurring in other systemic autoimmune rheumatic diseases [18]. In 2011, Salajegheh et al. showed that autoantibodies targeting a ~44 kDa human muscle protein (referred to as Mup44) occur in the serum of 52% of patients with sIBM (*n* = 25) with 100% specificity for the disease [19]. In 2013, two independent studies identified Mup44 as cytosolic 5′-nucleotidase 1A (cN1A), which is an enzyme highly expressed in skeletal muscle that catalyzes the dephosphorylation of adenosine monophosphate into adenosine and phosphate [20,21]. Even if absent or extremely rare in healthy controls, antibodies against cN1A have also been detected in primary Sjögren’s syndrome (pSS) and systemic lupus erythematosus (SLE), making them not specific for sIBM [22,23]. Nevertheless, anti-cN1A antibodies are considered a valuable diagnostic biomarker for sIBM because they occur in 33 to 76% of patients with sIBM and in less than 5% of patients with PM, DM, or non-autoimmune neuromuscular diseases [23]. It has been reported that the specificity of anti-cN1A to distinguish sIBM from other IIM, neuromuscular diseases, and autoimmune disorders ranges between 87 and 100% and between 74.6 and 92% to discriminate sIBM from other types of IIM [24]. Contradictory observations have been made regarding the association of anti-cN1A antibodies with a more severe phenotype and increased mortality risk independent of age, gender, comorbidities, and the presence of dysphagia [20,23,24,25,26,27,28,29,30,31]. Even though there is no cure for sIBM, antibodies against cN1A are helpful in the diagnostic workup to reduce misdiagnosis with PM and consequent administration of steroids that worsen sIBM progression after discontinuation [10,15].

### 2.3. Magnetic Resonance Imaging (MRI)

In recent years, muscle MRI has emerged as an important tool in the diagnosis of muscle disorders due to its ability to highlight affected muscle by revealing atrophy, fat infiltration and signs of inflammation [32]. In sIBM, MRI changes have been reported in both upper and lower limbs, are more severe in distal segments, and usually have an asymmetric pattern [32,33,34,35]. Fat infiltration is commonly seen in MRI scans [33,35,36,37]. It has been observed a distinctive MRI pattern that can discriminate between sIBM and other myopathies, namely the relative sparing of the rectus femoris, hamstrings, and adductor muscles and the extent and asymmetry of fatty infiltration, mostly seen in the adductor magnus [38]. Furthermore, flexor digitorum profundus seems to be involved in sIBM patients on imaging even before its weakness becomes clinically detectable [38].

### 2.4. Pathological Characteristics

**Figure 1:**
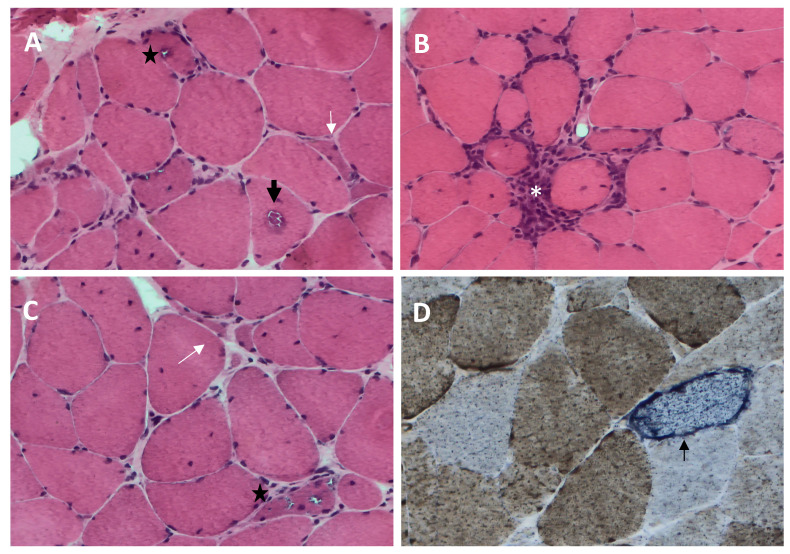
Histopathological features of sporadic inclusion body myositis (sIBM) muscle biopsy ((**A**–**C**), hematoxylin and eosin; (**D**), cytochrome c oxidase (COX)-succinate dehydrogenase): endomysial inflammatory infiltrate that invades nonnecrotic muscle fibers (white asterisk, (**B**)), rimmed vacuoles within muscle fibers (black star, (**A**,**C**)), eosinophilic cytoplasmic inclusion (black arrow, (**A**)), ragged red fiber and COX-negative fibers (thin black arrow, (**D**)), and angulated muscle fibers (white arrow, (**A**,**C**)).

sIBM muscle biopsy shows both degenerative and inflammatory features (Figure 1). Histopathological changes detectable by light microscopy include the following.

(1) Rimmed vacuoles, namely irregular vacuoles of variable size and shape, bordered by basophilic granular deposits that occur in nonnecrotic muscle fibers. Rimmed vacuoles can be a rather rare finding and are visible in 0.4–6.4% of the fibers [9];
(2) Eosinophilic cytoplasmic inclusions visible at hematoxylin and eosin (H&E) and modified Gomori trichrome staining [17];
(3) Cytoplasmic accumulation of aggregated/misfolded proteins, referred to as amyloid deposits or protein inclusions, which occur in 60 to 80% of the sIBM vacuolated muscle fibers, usually in non-vacuolated areas of the fiber [1,17]. These β-pleated-sheet amyloid inclusions are detectable by fluorescence-enhanced Congo red staining. Several proteins have been found within these aggregates, including amyloid-β precursor protein (AβPP), amyloid-β (Aβ), phosphorylated-tau (p-tau), and ubiquitin, to name a few [8]. Notably, TDP-43 immunopositivity has been reported in over 60% of sIBM patients and is considered a hallmark of sIBM pathology [39,40];
(4) Endomysial lymphocytic infiltrates consisting predominantly of macrophages and CD8^+^T cells that invade nonnecrotic muscle fibers expressing MHC class I on the sarcolemma [17];
(5) Mitochondrial abnormalities consisting of the abnormal proliferation of mitochondrial leading to ragged red fibers (RRFs, muscle fibers containing excessive mitochondrial proliferation) and the impairment of mitochondrial function, as shown by increased cytochrome c oxidase (COX)-negative fibers [41,42];
(6) Angulated muscle fibers of small caliber suggesting a neurogenic process [43].

Ultrastructural analysis reveals characteristic degenerative changes such as the following:

(1) Tubulofilamentous inclusions of 15–21 nm in the form of pair helical filaments (PHF) containing p-tau and located in the sarcoplasm and/or nucleus [1,17];
(2) Cytoplasmic 6–10 nm amyloid-like filaments containing Aβ, deposits of flocculomembranous and amorphous material [1].

### 2.5. Correlation of Pathological Features with Clinical Progression and Laboratory Findings

Overall, only a few studies have attempted to correlate specific clinical and/or laboratory findings with muscle histopathological changes. A recent retrospective study on 50 sIBM patients documented a strong and statistically significant positive correlation of endomysial inflammation with the severity of dysphagia and, conversely, a negative correlation with the modified Rankin scale (mRS) whereas the degenerative features such as rimmed vacuoles and congophilic inclusions showed no correlation with any of the clinical measures including muscle strength sum score, quadriceps strength, mRS and severity of dysphagia [44]. Varying and conflicting observations have been made regarding the association of anti-cN1A antibodies with pathological features, thus anti-cN1A antibodies correlated with fewer rimmed vacuoles [45], more cytochrome oxidase negative myofibers [25,46], a higher number of regenerating fibers [44], or auto-aggressive inflammation [47]. CK levels correlated with a broad spectrum of histological findings, including muscle fiber necrosis, regeneration, endomysial and perimysial inflammation, and increased endomysial connective tissue [44].

## 3. Degenerative Processes in sIBM Pathogenesis

**Figure 2:**
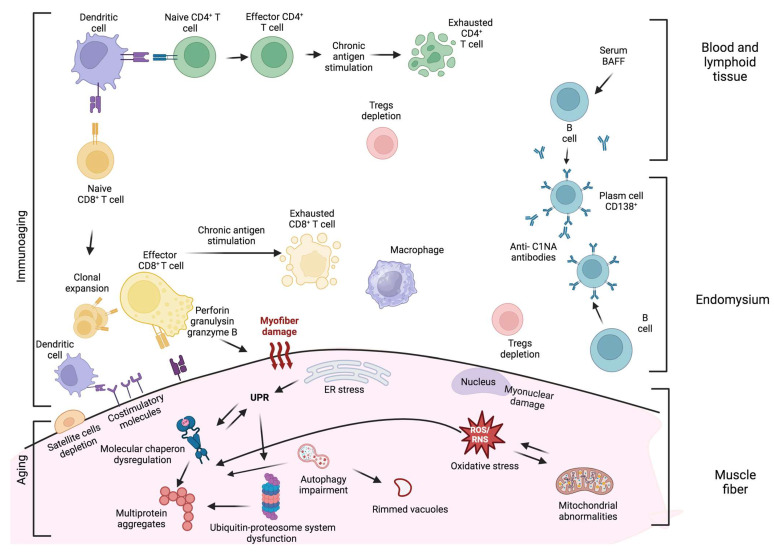
Schematic representation of the main molecular mechanisms contributing to the pathogenesis of sporadic inclusion body myositis (sIBM).

The skeletal muscle of patients with sIBM is characterized by several degenerative processes, including cytoplasmic accumulation of misfolded/toxic proteins, vacuolization, impairment of the ubiquitin–proteasome system and autophagy, oxidative and nitrative stress, endoplasmic reticulum stress, mitochondrial dysfunction, aging, and abnormalities of protein transcription and processing [8] (Table 1 and Figure 2).

### 3.1. Protein Aggregation

Protein aggregation represents the main degenerative feature of sIBM. Protein misfolding/unfolding was initially discovered with the detection of cytoplasmic congophilic deposits indicating the presence of amyloid inclusions [48]. This observation was followed by the identification of Aβ protein, tau, and ubiquitin in the aggregates [49,50,51,52,53]. Aβ peptides, mainly Aβ40 and Abβ42, are produced from AβPP via sequential proteolytic cleavage by the β-secretases β-site of the APP cleaving enzyme 1 and 2 (BACE1 and BACE2) and the γ-secretase complex [54]. It has been shown that among the different forms of Aβ proteins, the more cytotoxic Aβ42 is predominantly aggregated in sIBM muscle fibers and occurs in the form of oligomers [55]. Increased plasma Aβ42 was also reported in patients with sIBM [56]. Moreover, the proteases involved in AβPP processing were found to be significantly increased and/or accumulated in sIBM [53,55,57,58,59]. Phosphorylated tau has also been detected in cytoplasmic aggregates where it was found to colocalize with kinases involved in its phosphorylation, such as extracellular signal-regulated kinase (ERK), cyclin-dependent kinase 5 (CDK5), glycogen synthase kinase 3β (GSK-3β), and casein kinase 1 [60,61,62,63,64,65].

The detection of Aβ and p-tau initially received a lot of attention due to their already-known involvement in Alzheimer’s disease. Nevertheless, over decades, several other proteins have been found to abnormally accumulate in the cytoplasm of sIBM muscle fibers in the form of aggregates/inclusions, and many of them are linked to different neurodegenerative disorders. These include proteins prone to unfold/misfold, such as α-synuclein, presenilin 1, and prion, proteins involved in endoplasmic reticulum (ER) stress (GRP78 and GRP94) and oxidative/nitrative stress (nitrotyrosine, NOS and SOD1), protein-folding homeostasis (heat shock proteins, 26S proteasome components, ubiquitin, and p62/SQSTM1), and RNA metabolism (RNA polymerase II, FUS, TDP-43, VCP, c-Jun, NFkB hnRNPA1, hnRNPA2/B1, hnRNPC1/C2, and hnRNPH) [8,66,67,68].

Even though the list of proteins that are abnormally accumulated in sIBM aggregates has been growing over the years, the pathogenic events that drive the abnormal cytoplasmic localization and aggregation of these proteins, as well as their relationship with the immune component of sIBM still remain unknown. A combination of processes likely contributes to the protein aggregation: these include increased protein translation and specific post-translational modification, impaired protein disposal via the ubiquitin–proteasome system (UPS) and autophagy pathway, and altered cellular environment due, for example, to oxidative stress and aged milieu that promote damage and misfolding/unfolding of proteins and interfere with protein quality control systems [40].

### 3.2. Impairment of Ubiquitin–Proteasome System (UPS) and Autophagy

In the cell, several events can trigger protein misfolding, such as protein mutation, harsh cellular environment, and metabolic stress [69,70]. Misfolded proteins can have deleterious consequences for the cells due to the loss of specific function/s or the acquisition of toxic activities and the tendency to form insoluble aggregates [69,70]. Cells are equipped with protein quality control mechanisms that aim to maintain protein homeostasis (or proteostasis) by refolding, degrading, or segregating misfolded proteins [69,70]. Molecular chaperons constitute a group of proteins that bind to unstructured proteins, including nascent polypeptides on ribosomes and abnormally exposed hydrophobic regions, to prevent protein misfolding and facilitate the acquisition of correct protein conformation [71]. A few studies are reported in the literature that suggest a possible alteration of molecular chaperones in sIBM [72,73]. HSP70 immunopositive inclusions have been observed in sIBM muscle [72]. Specifically, HSP70 immunoreactivity was found to be colocalized with Aβ and p-tau signal, mostly in vacuolated muscle fibers. It has also been found that the HSP70 protein is upregulated in sIBM muscles and co-immunoprecipitates with Aβ [72]. This observation has been interpreted as a possible role of HSP70 in Aβ folding, although it cannot be excluded that this interaction is the result of unspecific binding due to protein unfolding/misfolding. αB-crystallin (αBC), a member of the small heat shock protein family, has also been found to be accumulated in sIBM muscle fibers and in other muscle diseases [73,74]. Evidence for the association between αBC with AβPP and Aβ oligomers has been shown in both sIBM muscle and in AβPP-overexpressing cultured human muscle fibers. It has been proposed that the binding of αBC to Aβ oligomers might hinder the segregation of Aβ oligomers into non-toxic aggregates and, therefore, prolong their toxic effects [72,74].

The failure of molecular chaperones to assist protein folding results in the presence of unfolded/misfolded proteins in the cell that can have toxic and harmful effects. In addition, other types of protein damage resulting, for example, by oxidation, accumulation of proteins in insoluble aggregates can be cytotoxic. Therefore, unfolded/misfolded, damaged, and aggregated proteins need to be eliminated to avoid or minimize cellular toxicity. Two major systems are responsible for protein degradation, namely the ubiquitin–proteasome system (UPS) and the autophagy pathway. Alterations in both processes have been described in sIBM and are considered important players in the pathogenesis of the disease [40].

The UPS consists of multiple components that act synergistically to remove aberrant proteins [75]. E1 ubiquitin-activating enzymes, E2 ubiquitin-conjugating enzymes, and E3 ubiquitin–protein ligases act sequentially to tag proteins that need to be eliminated with multi-ubiquitin chains [75]. Shuttle factors are then responsible for the delivery of ubiquitinated proteins to the 26S proteasome, the molecular machine responsible for the degradation [75]. The 26S proteasome is a multiprotein complex composed of a 20S catalytic subunit and one or two 19S regulatory subunits [75]. The regulatory complex unfolds the ubiquitin-conjugated proteins so that they can enter the 20S core, where they are subjected to different proteolytic activities of various subunits of the 20S core [75]. Abnormalities in the UPS in sIBM are supported by the increased expression and accumulation of 26S proteasome subunits and decreased activity of proteasomal proteolytic enzymes [76,77]. The impairment of the UPS is also supported by the observation that multiprotein aggregates in sIBM have features of aggresome, an extreme form of protein aggregates [78]. Aggresome formation is a protective mechanism activated under chronic UPS inhibition that aims to sequester misfolded proteins to prevent their possible toxic effects and eliminate them via autophagy [71].

Autophagy is an intracellular catabolic pathway through which components that cannot be degraded by the UPS, such as part of the cytoplasm, protein aggregates, malfunctioning organelles, or invading pathogens, are eliminated and recycled by the cell [79]. These components are first segregated in double lipid bilayer structures known as autophagosomes and then delivered to lysosomes to be degraded by lysosomal hydrolases into macromolecules such as aminoacidic, nucleotides, and sugars that can be reused by the cell [79]. Autophagy allows the elimination of damaged components and the maintenance of the proper number of functional organelles. Furthermore, the cell can exploit autophagy to self-digest some of its structure to survive under conditions of low nutrients and stress [79]. Several studies have provided evidence that impairment of autophagy contributes to the accumulation of proteins in the form of aggregates in muscle fibers of patients with sIBM. A histological hallmark of sIBM is the presence of cytoplasmic vacuoles. The autophagic nature of these vacuoles is supported by their positivity for acid phosphatase and other lysosomal enzymes, as well as by their content consisting of membranous debris that could arise from remnants of impaired organelles [80,81]. The first indication of possible involvement of autophagy in sIBM muscles came from the detection of increased mRNA or protein levels of some lysosome proteins, including M6PR, clathrin, and hApg5 and hApg12 in sIBM muscles [81]. Later, Atg8/LC3-positive autophagic vacuoles containing AβPP and Aβ were detected in muscle fibers from sIBM patients [82]. These AβPP/Aβ and Atg8/LC3 double-positive vacuoles were almost exclusively detected in degenerating muscle fibers and occurred in a subset of muscle fibers expressing major histocompatibility complex (MHC) class II and surrounded by CD4^+^ and CD8^+^ T cells. This finding indicates a possible involvement of autophagy in the generation of antigens for MHC presentation to invading T cells [82]. Other factors involved in autophagy have been found to be altered in sIBM muscles. Increased transcript and protein levels of p62/SQSTM1, a factor involved in targeting ubiquitinated proteins for proteasomal and lysosomal degradation, were detected in sIBM muscle and found to colocalize with p-tau positive inclusions. Based on the increase in p62 that occurs following pharmacological inhibition of proteasomal or lysosomal protein degradation, it has been proposed that defective protein degradation via these two processes might participate in sIBM pathology [83]. In support of this scenario, a decrease in the activity of the lysosomal enzymes cathepsin D and B was found in sIBM muscles [80].

Micro-tubule-associated protein 1 light chain 3B (LC3) is detected, under normal conditions, in the cytoplasm as LC3I forms. After induction of autophagy, LC3I is conjugated to phosphatidylethanolamine to generate LC3II, which is relocated to autophagosomes and autolysosomes and is therefore considered a marker of autophagy [84]. LC3II and a decreased ratio of phosphorylated p70S6 kinase to total p70S6 kinase were considered an indication of autophagy induction in sIBM muscles [80]. According to this interpretation, the accumulation of LC3II and its binding partner p62 in sIBM muscle fibers could be the result of increased autophagosome formation [85].

Like p62/SQSTM1, NBR1 is another carrier protein involved in the transport of ubiquitinated proteins for autophagic degradation. It has been found that NBR1 protein is increased and accumulated in sIBM muscle in aggregates immunopositive to p62, ubiquitin, and phosphorylated tau [86].

Increased expression levels of the autophagy markers Beclin 1, ATG5, and LC3 and of the endosomal marker clathrin were detected in sIBM muscle and attributed to increased autophagosomal and endosomal structure due to increased autophagosome formation and/or to impaired late steps of the autophagy pathway [87]. It has also been shown that Beclin 1 associates with p-tau in the sarcoplasm of s-IBM myofibers and that lymphocytes preferentially surround Beclin 1 fibers [87].

An upregulation of components of the chaperone-mediated autophagy (CMA), a form of lysosomal pathway responsible for the degradation of proteins carrying the KFERQ motif, has been reported in sIBM muscle [88]. Hsc70, involved in the recognition and binding of proteins carrying the KFERQ motif, and LAMP2A, which binds the Hsc70-chargo protein complex at the level of the lysosome, unfold and transfer target proteins within the lysosome, are both increased in sIBM muscle [88]. Interestingly, α-synuclein, which contains a KFERQ motif and is a CMA substrate, was found to be physically associated with LAMP2A and Hsc70 [88]. These observations might indicate that CMA is induced in sIBM muscle to remove misfolded proteins and avoid the formation of insoluble protein aggregates. However, since these proteins are targeted to defective lysosomal structure, as suggested by the decreased lysosomal enzyme activity, they are not properly cleared but accumulated in aggregates [88].

Overall, these studies support that impaired proteostasis occurs in sIBM and contributes to protein accumulation by reducing protein disposal and recycling.

### 3.3. Endoplasmic Reticulum (ER) Stress and Unfolded Protein Response (UPR)

The endoplasmic reticulum (ER) plays a critical role in the processing, folding, post-translational modification, and exporting of newly synthesized proteins into the secretory pathway [89,90]. Under certain conditions, ER protein folding capacity becomes saturated, and unfolded/misfolded proteins accumulate in the ER lumen, compromising the ER homeostasis, a state referred to as ER stress [89,90]. In an attempt to cope with unfolded/misfolded protein loading, the cell triggers a set of signaling pathways, collectively known as the unfolded protein response (UPR), that ultimately regulate gene expression to increase ER abundance and protein folding machinery and to repress protein synthesis [89,90]. UPR involves three major signaling pathways, each of which is activated by specific ER stress sensor proteins that reside in the ER membrane, such as ATF6 (activating transcription factor 6), IRE1 (inositol requiring enzyme 1), and PERK (double-stranded RNA-activated protein kinases (PKR)-like ER kinase) [89,90]. An additional pathway to relieve ER stress is ER-associated degradation (ERAD), by which unfolded/misfolded proteins undergo retrotranslocation into the cytosol to be degraded by UPS [90].

ER stress, UPR activation, and ERAD induction have been documented in muscle fibers of sIBM patients, highlighting the significant role of ER stress in the pathogenesis of sIBM. Increased expression of several ER chaperones, including calnexin, calreticulin, BiP/GRP78, GRP94, and ERp72, have been found in muscle specimens of patients with sIBM [91]. Furthermore, both in sIBM muscle and in the AβPP-overexpressing cultured human muscle fibers, those chaperones physically interact with Aβ, suggesting their possible role in Aβ folding process and/or in Aβ protein clearance [91]. The presence of the processed ATF6 N-terminal fragment, which mediates the upregulation of UPR target genes, including BiP/GRP78, the production of the alternatively spliced form of XBP1, which leads to the transcriptional activation of chaperones and folding proteins involved in ERAD, the increased expression of ATF4 protein, and the augmented levels of CHOP mRNA, which is regulated by the oligomerization of PERK and subsequent phosphorylation of the transcription factor eIF2α, were detected in muscle biopsies of sIBM patients, suggesting that all the three main UPR pathways are activated in sIBM [92].

Many other proteins implicated in ER stress have also been found to be altered in sIBM muscle. Upregulation at mRNA and protein level of Herp (homocysteine-induced ER protein), an ER stress-inducible protein normally localized to the ER membrane, and its focal accumulation in the cytoplasm of affected fibers together with Aβ, the ER chaperone BiP/GRP78, and the 20S β2 proteasome subunit was detected in the muscle of patients with sIBM [93]. Furthermore, Herp was found to be overexpressed both at mRNA and protein levels in ER stress-induced cultured human muscle fibers, where the protein presented a diffuse staining, and increased at protein level in proteasome-inhibited cultured human muscle fibers, where it forms cytoplasmic aggregates [93]. These observations led the authors to speculate that the increased expression of HERP is induced in sIBM muscle fibers by both ER stress and proteasome inhibition to stimulate the ER folding capacity and misfolded protein removal [93].

In cultured human muscle fiber, ER stress has been demonstrated to regulate the expression of MstnPP (myostatin precursor protein) via the transcription factor NF-κB that becomes activated during UPR [94]. In skeletal muscle, MstnPP is post-translationally processed primarily into myostatin (Mstn), a member of the transforming growth factor beta (TGF-β) superfamily that negatively regulates muscle development and growth. Increased expression of MstnPP, at both the mRNA and protein levels, was observed in the muscle of patients with sIBM [95,96]. Myostatin dimers were detected in sIBM muscle and found to colocalize and physically interact with Aβ/AβPP [95,96]. These findings led to the hypothesis that an aberrant aggregation of MstnPP/Mstn contributes to muscle fiber atrophy and weakness in patients with sIBM.

### 3.4. Mitochondrial Abnormalities

Mitochondrial alterations have been widely described in muscles of patients with sIBM. These alterations include mitochondrial proliferation, COX-negative fibers, and mitochondrial DNA (mtDNA) changes and occur with a greater extent and frequency in sIBM patients compared to healthy age-matched controls [97].

Mitochondria proliferation is detectable by the modified Gomori trichrome stain, which highlights the accumulation of abnormal mitochondria (red stain) in the subsarcolemmal region of the muscle fibers that look overall irregular in shape and are, therefore, referred to as “ragged red fibers” (RRFs). In patients with sIBM, RRFs represent 1% of the total fibers, whereas in the elderly, RRFs occur with a frequency of about 0.3% [97]. Accumulations of mitochondria might represent a compensatory response to the impaired functionalities of these organelles. An increased percentage of COX-negative fibers in sIBM patients compared to healthy aged-matched controls has been reported in several studies [98,99]. Reported frequencies of COX-negative fibers range from 0.5% to 5% of total fibers in sIBM muscles, whereas they are only occasionally observed in healthy subjects with similar ages [98]. Impaired oxidative phosphorylation (OXPHOS) has been further supported by biochemical studies that detected a 30% decrease in COX activity, confirming the COX deficit observed by histological analysis [99].

An impairment of mitochondrial function has also been detected in primary myoblasts isolated from patients with sIBM. sIBM primary myoblasts displayed reduced oxygen consumption rate (OCR) and intracellular ATP but increased extracellular acidification rate (ECAR) compared to normal myoblasts, indicating the activation of glycolysis to compensate for the reduced oxidative phosphorylation [100].

Abnormalities in the expression of some of the mitochondrial respiratory complexes have been observed in sIBM muscles. A reduction in the protein levels of complex I, III, and, with more variability, complex IV, which are partly encoded by mtDNA, was documented in sIBM muscle, supporting a functional alteration of the respiratory chain [101].

Mitochondrial function is tightly linked to mitochondrial dynamics and morphology. Abnormalities in mitochondria structure have also been observed by ultrastructural analysis on sIBM muscles [98,100]. Structural alterations include mitochondria enlargement, loss of cristae, and paracrystalline inclusions [98,100]. Some of these structural abnormalities might result from disturbances of the mitochondrial dynamics occurring in sIBM muscle. Alteration of the expression of mitofusins (Mfn), which control fusion of the outer mitochondrial membrane, and of optic atrophy 1 (OPA1), which regulates fusion of the inner mitochondrial membrane, have been found in sIBM and contribute to the abnormalities of the mitochondrial network [99]. A decrease in Opa1 and Drp1 mRNA levels was reported in sIBM patient myoblasts compared with normal control myoblasts, whereas the expression levels of mitofusin 1 (Mfn1) and mitofusin 2 (Mfn2) were found to be unchanged [100]. Time-lapse imaging of mitochondria revealed decreased mitochondrial mobility in primary myoblasts obtained from sIBM patients compared to myoblasts from healthy subjects [100]. Alterations in mitochondrial biogenesis in sIBM are suggested by the altered expression of important regulators of this process [97,102]. The non-coding microRNAs (miRNAs) miR-133 is markedly reduced in sIBM, whereas gene expression of peroxisome proliferator-activated receptor γ coactivator-1α (PGC-1α) is increased in sIBM compared to control muscles [97,102]. Interestingly, metabolomics studies performed on peripheral blood revealed that patients with sIBM share metabolic changes with subjects affected by primary mitochondrial myopathies [103]. Notably, a greater degree of lymphocytic and macrophage infiltration has been observed in sIBM muscle fibers with more respiratory chain dysfunction. A strong positive correlation between the degree of inflammation, mitochondrial changes, and atrophy has been found, suggesting that mitochondrial dysfunctions have a role in sIBM progression [104].

As part of mitochondrial pathology, a remarkable mtDNA depletion and the presence of mtDNA alterations have been found in sIBM [42,98,100,101,105,106]. mtDNA deletions have been detected in muscles from sIBM patients with different techniques [98,105]. PCR and Southern blot analysis detected the occurrence of multiple mtDNA deletions in muscle tissue of IBM patients [98,105]. In situ hybridization using different mtDNA probes revealed that deleted mtDNA accumulates in COX-deficient muscle fibers in patients with sIBM [98]. PCR analysis performed on isolated single muscle fibers has detected mtDNA with only one type of deletion in each COX-deficient muscle fiber, suggesting that clonal expansion of deleted mtDNA might occur in each COX-negative fiber [105]. The 4977 bp “common deletion”, which deletes between nucleotides 8470 and 13,447 of the human mtDNA, is frequently detected COX-deficient fibers in sIBM muscles [42,105]. This mutation leads to the loss of all or part of the genes encoding four subunits of complex I, one subunit of complex IV, two subunits of complex V, and five tRNA genes and, as expected, has a major impact on mitochondrial functionality [107].

Recently, deep sequencing of mtDNA by whole genome sequencing (WGS) has led to the identification of somatic mtDNA variants (deletions, duplications, and single nucleotide variants) [101]. An increase in all these mtDNA alterations has been found in sIBM muscle compared to age-matched controls [101]. mtDNA copy numbers were also increased in muscle samples from sIBM patients, as reported in other studies [100,101]. Novel variants in nuclear genes involved in mtDNA maintenance have been found in sIBM. New variants of POLG and C10orf2 and a significantly higher frequency of an RRM2B variant were reported in IBM patients, suggesting that alterations in mechanisms involved in mtDNA maintenance might contribute to the disease [41].

### 3.5. Oxidative Stress

The possible causative role of oxidative and nitrative stress in sIBM pathogenesis was first proposed by Askanas and Engel [108]. Nitric oxide (NO) is a short-lived free radical that plays a significant physiological role in different biological processes, including vasodilation, antimicrobial activity of activated macrophages, and transcriptional and post-transcriptional regulation of genes [109]. NO generation is catalyzed by three distinct isoforms of nitric oxide synthase (NOS): endothelial (eNOS), neuronal (nNOS), and inducible (iNOS) [110]. Nitric oxide (NO) can react with superoxide, a biologically relevant reactive oxygen species (ROS), to generate peroxynitrite, a highly reactive nitrogen species (RNS), which in turn leads to the conversion of tyrosine to 3-nitrotyrosine (3-NT), either free or part of a polypeptide chain [109]. The nitration of tyrosine can compromise the functional and/or structural integrity of target proteins [109]. The occurrence of nitrative stress in sIBM is supported by the abnormal accumulation of nitrotyrosine and of two NOS isoforms, nNOS and iNOS, in vacuolated fibers of sIBM muscle [111].

Since superoxide can be toxic, mammalian cells contain an enzymatic protective system against ROS-mediated damage, which mainly includes manganese superoxide dismutase (MnSOD) and copper–zinc superoxide dismutase (CuZnSOD). MnSOD, located in the mitochondria, and CuZnSOD, located in the cytoplasm, are scavenging enzymes that transform superoxide anions into hydrogen peroxide [112]. MnSOD and CuZnSOD were found to be increased and accumulated in vacuolated muscle fibers of patients with sBM, but while MnSOD colocalized with nitrotyrosine and occasionally with p-tau, CuZnSOD was associated with ubiquitin, suggesting that the two enzymes could have a different role in fiber protection [113,114]. It has been therefore hypothesized that while MnSOD exerts a protective effect in muscle fibers against oxidative and nitrative stress, CuZnSOD participates in ubiquitination and clearance of inclusions in affected fibers [113,114]. Oxidative stress could also act as a key upstream trigger of AβPP overexpression in sIBM muscle via the upregulation of transcription factor NF-kB and redox factor-1 (Ref-1) [111,115].

Other factors that exert a protective function against the harmful effects of oxidative stress have been found in sIBM muscle. Increased mRNA and protein levels of insulin-like growth factor I (IGF-I), a pleiotropic growth factor with endocrine, paracrine, and autocrine functions, and members of one of two main IGF-I signaling pathways, namely phosphoinositide-3-kinase (PI3K) and Akt, were detected in affected but not regenerating muscle fibers containing Aβ inclusions [116]. Based on the upregulation of IGF-I mRNA and protein that occurs in primary muscle cultures treated with Aβ(25–35) peptide, it has been postulated that IGF-I overexpression represents a protective response in vulnerable fibers to Aβ toxicity and that oxidative stress could play an important role in leading to increased expression of IGF-I [116]. DJ-1 is a small ubiquitously expressed protein that dimerizes under physiological conditions and plays several cellular functions, including antioxidant response, chaperone activity, autophagy regulation, mitochondrial homeostasis, transcription regulation, and neuroinflammation reduction [117]. DJ-1 was reported to be increased at both mRNA and protein levels and highly oxidized in the muscle of patients with sIBM [118]. It has also been shown that DJ protein forms cytoplasmic aggregates only in a small percentage of abnormal muscle fibers and is overexpressed as a 46 kDa dimer in addition to the 23 kDa monomer in the soluble fraction of sIBM muscle, while the mitochondrial-enriched fraction contains only monomer. These observations have been interpreted as a possible role of DJ-1 in counteracting oxidative stress and mitochondria dysfunction in abnormal muscle fibers [118].

It is conceivable that multiple mechanisms can lead to the generation of ROS/RNS and oxidative/nitrative stress in sIBM; one potential explanation is that mitochondrial dysfunction documented in the muscle of sIBM patients causes increased ROS production with consequent oxidative stress and cellular damage.

### 3.6. Nuclear Degeneration

The possible involvement of the nuclear compartment in sIBM has been reported in the literature [119,120]. Changes in the nuclear shape, the release of filamentous structures from the nucleus to the cytoplasm, and alterations in the heterochromatin were observed in sIBM muscles and suggested that nuclear damage is involved in the disease [2,121,122,123]. The detection of the nuclear proteins emerin and lamin A/C within the rimmed vacuoles and the nuclear acid binding proteins, such as TDP-43 and other hnRNPs, in the sarcoplasm further support the idea that myonuclear damage contributes to protein mislocalization and accumulation in the cytoplasm [68,124,125]

Nevertheless, the exact nature of these nuclear alterations and their role in sIBM pathogenesis remain still unknown.

## 4. Aged Skeletal Muscle Milieu

Aging is the progressive decline in cellular and physiological functions over time that leads to reduced survival [126]. Aging has been identified as a risk factor for a variety of human diseases, including cancer, cardiovascular, and neurodegenerative diseases [126]. sIBM mainly affects the elderly, with age at diagnosis over 50, strongly pointing to a key role of age-associated changes in the onset and progression of the disease.

Skeletal muscle, like all other tissues, undergoes age-dependent structural and biochemical changes that progressively impact its physiological functions. During aging, skeletal muscle experiences a reduction in protein synthesis [127], infiltration of adipose tissue and fibrosis [128], misregulation of proteostasis [129], dysfunctional autophagy, and mitophagy [130], mitochondrial alterations [131], altered nuclear shape and spatial disorganization of nuclei [132], changes in nuclear-cytoplasmic signaling [133], decrease satellite cells number and function [134], increased ROS generation [135], DNA damage [136], and chronic inflammation [137]. Many of the processes contribute to the age-dependent decrease in myofiber size that causes loss of muscle mass and strength in older adults, a condition known as sarcopenia [138].

Several alterations affecting skeletal muscle during aging have been observed in the skeletal muscle of patients with sIBM, usually to a greater extent than in the skeletal muscle of aged-matched healthy subjects. Aging is associated with impaired protein homeostasis or proteostasis [126]. Proteostasis is maintained via mechanisms that ensure adequate proper synthesis, folding, and degradation [69]. These mechanisms work together to ensure a continuous supply of functional proteins and prevent the accumulation of damaged and malfunctioning proteins. A decrease in the production of protein chaperones and in the activity of the autophagy-lysosomal and ubiquitin–proteasome systems are responsible for the loss of protein homeostasis during aging. Impaired proteostasis has been documented in sIBM and might contribute to protein aggregation [139].

Altered nuclear architecture and changes in nucleocytoplasmic signaling have been reported in skeletal muscle during aging [140]. Structural alterations have been observed in sIBM myonuclei, suggesting that impairment of nuclear architecture and function due to aging may contribute to sIBM pathology [122,123].

Alterations in satellite cells (SCs) have been described during aging [141]. Despite an increased number of Pax7^+^ SCs having been reported in sIBM muscle specimens, indicating an ongoing repair process, the expression of the myogenic regulatory factors MyoD and myogenin was found to be altered in the disease. These observations suggest that there might be a delay and/or decrease in later steps of SC activation and myoblast proliferation, which could be due to replicative senescence of SCs after repeated cycles of activation rather than a depletion of the pool of satellite cells [142,143]. These abnormalities in the myogenic program could contribute to poor muscle repair in sIBM.

mtDNA is particularly susceptible to mutating during aging due to the highly oxidative mitochondrial environment, lack of protection from histone proteins, and low efficiency of mtDNA repair mechanisms compared to genomic DNA [144]. As a result, the somatic mutation rate of mtDNA is estimated to be 10 to 20 times higher than that of nuclear DNA [145]. Oxidative damage to mtDNA has been shown to occur during aging in skeletal muscle, leading to the accumulation of mtDNA mutations over time [146,147,148,149]. The 4977-bp “common deletion” is the most frequent age-associated mtDNA mutation and is also found in sIBM muscles [150]. This and other age-related mtDNA mutations lead to defective respiratory enzymes and, consequently, reduced oxidative phosphorylation and increased ROS production [149]. These alterations trigger oxidative stress and oxidative damage of the mitochondria, which contribute to further damaging mtDNA, perpetuating a vicious cycle.

It is emerging that, not surprisingly, muscle cells are not the only cell type to be affected by aging in the skeletal muscle milieu. In a recent study, RNA sequencing performed on single nuclei isolated from muscle biopsies of patients with sIBM led to the identification of a population of senescent fibro-adipogenic progenitors (FAPs) that by secreting specific immunomodulatory cytokines and growth factors might contribute to inflammation and fibrosis in sIBM [151]. As discussed later, the aging of immune cells likely contributes to sIBM and poses further challenges for the treatment of this disease.

Aging is characterized by changes in many molecular and cellular processes [126]. Although these age-associated alterations and the establishment of an aged milieu are clearly part of sIBM pathology, their contribution to sIBM pathogenesis remains poorly understood.

## 5. Inflammation in sIBM Pathogenesis

The involvement of the immune system in sIBM is supported by the presence of endomysial immune cell infiltration and circulating autoantibodies. Although the role of inflammation in the pathogenesis of sIBM has been under discussion for a long time, many studies have provided data to support the involvement of both innate and adaptive immunity in the disease (Table 1 and Figure 2).

### 5.1. T Cells

Infiltrating immune cells are mostly represented by cytotoxic CD8^+^T cells that surround non-necrotic muscle fibers and are often localized in proximity to MHCI-expressing myofibers [17]. It has been estimated that CD8^+^ T cells are about fivefold more abundant than CD4^+^ T cells in sIBM muscle [152]. Several studies have shown that CD8^+^ T cells have restricted expression of T cell receptor (TCR) V*α*/Vβ genes in the blood and muscle of sIBM patients and are clonally expanded, suggesting that muscle-specific antigen drives the inflammatory response in the disease [153,154,155,156,157,158,159,160,161]. Interestingly, the expression of Vβ genes was found to be different in muscle-infiltrating lymphocytes and peripheral blood, indicating that T cells expand in situ in response to antigens presented by MHCI-expressing muscle cells, or they are selectively recruited to the muscle [156,157]. Identical T cell clones have been found in different muscles and persist for years, indicating that they continuously react to muscle antigens, perpetuating the inflammatory response over time [155,162].

T cell activation requires antigen presentation via MHC and the interaction between CD28 on T cells with costimulatory molecules on antigen-presenting cells (APCs). Expression of the costimulatory molecules BB-1, ICOS, and B7-H3 by muscle fibers has been demonstrated in sIBM and suggests that myofibers may present the antigen and activate T cells expressing the appropriate TCR [163,164,165,166].

Regarding phenotypic and functional features, infiltrating CD8^+^ T cells express markers of highly differentiated effector cells, have proinflammatory and cytotoxic features, and have minimal or no proliferative ability [158,167,168,169,170]. Specifically, expression of the cytolytic molecules perforin, granulysin, and granzyme B, and natural killer (NK) markers CD16 or CD56, have been detected in CD8^+^T cells invading skeletal muscle, indicating they share functional and phenotypic features of NK cells [165,171,172,173]. The occurrence of Th1 immune response has been supported by increased expression levels of the cytokines and chemokines CXCL-9, CXCL-10, IL-12, CCL-2, and IL-1RA and by a higher number of IFN-γ expressing CD8^+^CD28^−^ T cells in the blood of patients with sIBM [169]. These findings indicate that cytotoxic T cells respond to yet unknown muscle antigen/s and contribute to the myofiber damage that occurs in sIBM [9].

On the other hand, several observations show that, in sIBM, T cells are highly differentiated and have reduced effector functions. In this regard, it has been found that peripheral blood and muscle infiltrating T cells are effector memory T (T_EM_) cells and terminally differentiated effector memory T (T_EMRA_), as indicated by the lack of expression of CD28 and by the acquisition of CD224 and CD57 expression [9,169,174]. Loss of CD28 and gain of CD57 in T cells are known to be associated with decreased proliferative capacity, terminal differentiation, senescence, and clonal exhaustion, a state occurring with chronic antigen stimulation [175,176]. Exhausted T-cells have decreased ability to respond to cytokines, have lost their effector functions, and have upregulated inhibitory receptors (IRs) such as programmed cell death protein1 (PD-1), lymphocyte activation gene-3 (LAG-3), T-cell immunoglobulin domain and mucin domain-3 (TIM-3), and T-cell immune receptor with Ig and ITIM domains (TIGIT) [174,177,178]. The main function of IRs is to negatively regulate the activation and the effector functions of T cells and are responsible for fine-tuning T cell activity [178]. IRs have a key role in controlling immune responses and establishing T cell tolerance [178]. The expression of PD-1 has been reported on T cells infiltrating sIBM muscle [179]. PD1 ligands PD-L1 and PD-L2 have been detected on infiltrating macrophages and on myofibers, respectively, and possibly form immunologic synapses with PD1 expressed by lymphocytes [179]. In support of an exhausted phenotype of T cells in sIBM, the mRNA levels of the exhaustion markers PD1, EOMES, TBX21, LAG3, CD244, TIM3, and KLRG1 were found to be increased in sIBM muscle [179]. Taken together, these data indicate that, in sIBM, persistent exposure to antigens drives muscle infiltrating T cells toward a state of exhaustion characterized by reduced or absent effector functions and proliferation ability. These observations suggest that the T cell-mediated cytotoxic damage of muscle fibers likely plays a role in the early stages of the disease before T cell exhaustion occurs.

Regulatory T cells (Tregs) represent a subpopulation of CD4^+^T cells which have the main function to suppress immune responses [180]. Their immune suppressive activity is crucial for keeping immune reactions against invading pathogens under control and maintaining immune tolerance toward self-antigens [180]. The decreased number and function of Tregs have been associated with loss of tolerance and autoimmune diseases [181]. Studies have demonstrated that Tregs are able to control local immune responses during regeneration after skeletal muscle injury [182,183]. Although Tregs suppression activity in sIBM was found to be normal, a reduction in their number was observed in the blood and skeletal muscle of sIBM patients and proposed as a factor contributing to the autoimmune response to skeletal muscle in this disease [169].

### 5.2. Plasma Cells and Antibody-Mediated Immune Response

Several studies have provided evidence for a muscle antigen-driven B- and plasma cell-mediated immune response and humoral immunity in sIBM. Differentiated CD138^+^ plasma cells, but not CD19^+^ or CD20^+^ B cells, and increased expression of immunoglobulin gene transcripts were detected in the skeletal muscle of patients with sIBM [184]. Analysis of the variable region of Ig H chain gene transcript obtained from sIBM muscles demonstrated that B cells and their descendent plasma cells undergo oligoclonal expansion, isotype switching, and accumulate somatic mutations [185]. These processes usually occur in secondary lymphoid organs where B cells migrate after exposure to antigens to maturate and differentiate into antibody-producing plasma cells [185]. Interestingly, it has been observed that in sIBM muscles, inflammatory infiltrates of T cells, B cells, plasma cells, and myeloid dendritic cells assemble in lymphoid structures that could support the in situ maturation of B cells into antibody-secreting plasma cells [186]. Furthermore, increased serum levels of B-cell activating factor (BAFF), a cytokine involved in the survival, maturation, and differentiation of B cells, were detected in the serum of patients with sIBM [187]. These findings indicate that local maturation of B cells to antibody-producing plasma cells occurs in the muscle of sIBM patients in response to muscle antigen/s [185]. In agreement with these observations, a recent study revealed the sIBM muscle B cell receptor (BCR) repertoire and showed that it has distinct features from those of other IIMs, indicating that it may originate from exposure to disease-specific antigens [188].

The presence of a humoral immune response in sIBM was further supported by the detection of antibodies against cN1A, as described previously in this manuscript. The pathogenic role of the anti-cN1A was supported by the finding that mice immunized with anti-cN1A IgG isolated from patients show increased sarcoplasmic aggregation of p62 and LC3, degenerative features of sIBM muscle [30].

Overall, these findings support that in sIBM, B cells become activated and mature into plasma cells that produce antibodies directed against a muscle antigen.

### 5.3. Macrophages and Dendritic Cells

Macrophages and myeloid dendritic cells (DCs) have also been detected in sIBM muscle [152,189]. Proteomic studies have shown that CD74, CD163, and STAT1 are increased in sIBM muscle, and immunohistochemical analysis revealed these molecules are enriched in macrophages [190]. Specifically, CD74 was found to be expressed in CD68^+^ macrophages and the sarcolemma. CD163 was detected in endomysial macrophages, and STAT1 was found in macrophages that displayed phagocytic activity [190]. The interferon-induced protein ISG15 and IRF8 were found to be strongly expressed on MHCII^+^ macrophages, indicating that these cells are in an activated state [190].

DCs are also infiltrating the sIBM muscle. A study of the two populations of DCs, namely myeloid DC (mDCs) and plasmacytoid DC (pDCs), revealed that mDCs surround and invade healthy myofibers [191,192]. The close colocalization of mDCs with T cells has been interpreted as a possible role of mDCs in antigen presentation to T cells [191,192].

These studies show that, despite the expression of MHC on muscle fibers of sIBM patients and their possible contribution to antigen presentation, professional antigen-presenting cells (APCs) also play a role in the pathogenesis of sIBM [193,194,195]. However, further efforts are needed to better characterize the phenotype and function of macrophages and dendritic cells in sIBM.

## 6. Aging of the Immune System and sIBM

Relevant to sIBM pathology, the immune system also undergoes notable changes with age, a process called inflammaging [196]. The development and function of the immune cells involved in the pathogenesis of sIBM have been shown to be affected by aging. The sIBM muscle is infiltrated by CD8^+^T cells and macrophages, immune cells that undergo important changes with age.

T cell development occurs in the thymus, where bone marrow-derived progenitors differentiate and undergo positive and negative selections to give rise to a functional and self-tolerant T cell repertoire [197]. During aging, T cell production in the thymus declines from approximately 16% to <1% over a lifetime [198,199]. In adults and the elderly, the decrease in the production of naïve thymic T cells is partially compensated by the proliferation of peripheral T cells, which, although sufficient to maintain a compartment of naïve CD4^+^ T cells, does not support an adequate pool of peripheral naïve CD8^+^ T cells that undergo greater severe decline during aging [200]. After antigen stimulation, naïve T cells become activated and differentiate into effector and memory T cells. Aging is accompanied by a shift in the composition of the T cell pool from naïve to memory T cells, accumulation of terminally differentiated T cells, restriction of the T cell receptor (TCR) repertoire, and decreased ability to generate a CD8^+^ T cell response [201,202,203,204]. The expression of CD28, which is involved in T cell survival, activation, and proliferation, by T cells declines during aging and contributes to reduced immune responses in the elderly [204]. These age-related changes in T cells have also been described in sIBM, and aging likely contributes to these alterations in the disease. Skeletal muscle Tregs are important for maintaining immune tolerance within skeletal muscle and controlling immune response and tissue regeneration after skeletal muscle injury [205]. Although an increase in Treg in secondary lymphoid organs occurs during aging, reduced recruitment of Treg cells into injured muscle and a decrease in their proliferation and retention at the injury site has been reported in old mice [206]. Interestingly, sIBM is associated with peripheral Tregs deficiency, which could promote autoimmune-like responses and halt skeletal muscle repair in this disease [169].

sIBM muscles are infiltrated by macrophages expressing type I/II interferon markers and are highly phagocytic [190]. A decline in the expression of surface molecules, including MHC-II and Toll-like receptors (TLRs), which impair their antigen presentation capacity, and a decrease in secretion of interleukin 6 (IL6) and tumor necrosis factor α (TNFα) after appropriate stimuli have been reported during aging [207]. Accordingly, a decline in proinflammatory macrophages, which have the main role in supporting the inflammatory response, and an increase in anti-inflammatory macrophages, which are involved in the resolution of inflammation and tissue healing, were observed in aged skeletal muscle [208]. Further characterization of the phenotype of skeletal muscle-infiltrating macrophage in sIBM could help to better understand their contribution to the disease.

Aging of the immune system likely influences the immune processes occurring in sIBM, and studies are needed to understand how aging alters the function of immune cells that infiltrate skeletal muscle and how these alterations contribute to the pathogenesis of sIBM.

## 7. Interplay between Degeneration and Inflammation

The occurrence of both degenerative and inflammatory processes in sIBM has given rise to the still-debated question of which of these two aspects drives the pathogenesis of the disease.

Inflammation has been proposed as the main driver of disease and responsible for the degenerative alterations [9]. The role of inflammation in causing degenerative changes in sIBM is supported by the observation that inflammatory cytokines induce the formation of protein aggregates and the expression of nitrative stress markers in cultured human myoblasts [209,210,211,212]. Studies in a transgenic mouse model of IBM have shown that inflammatory stimuli increase CD8^+^ T cell infiltration, AβPP and Aβ42 levels, and tau phosphorylation in skeletal muscle supporting the view of inflammation as the primary cause of sIBM [213].

On the other hand, the lack of response to immunosuppressive and immunomodulatory therapies in sIBM has been raised as a main argument against the primary role of inflammation in the disease [40]. In agreement with this interpretation, a recent study demonstrated that the deletion of T cells in an sIBM xenograft model does not rescue the degenerative features [214]. However, based on this finding, we cannot exclude the role of T cells in establishing the degenerative phenotype and that T cell depletion in the early stage of the disease may have a positive impact. Also, the exhausted phenotype of T cells infiltrating sIBM muscle could provide an explanation for the lack of response to immunosuppressive treatment and suggests that the cytotoxic T cell-mediated muscle fiber damage could play a role in early stages when the disease is not yet diagnosed. Recently, the involvement of the NLRP3 inflammasome has been reported in sIBM [215]. The inflammasome pathway can be triggered by different endogenous or exogenous signals leading to the secretion of pro-inflammatory cytokines via caspase-1 activation [216]. Activation of the inflammasome pathway has been detected in response to intracellular aggregation of proteins and occurring in other protein aggregation diseases [217,218]. Overexpression of NLRP3 inflammasome has been detected in muscle biopsies of patients with sIBM but not in patients with other IIM [215]. In sIBM muscles, inflammasome activation was found to positively correlate with pro-inflammatory and protein degradation markers, suggesting another possible crosstalk mechanism between inflammatory response and degeneration in sIBM [215].

Despite the well-recognized crosstalk between inflammation and degeneration in sIBM, it is not yet known which of these two processes is the *primum movens*. The causative relationship between the two aspects is also yet to be understood. Investigation of these aspects is not only scientifically intriguing but also paramount to developing effective therapies for this relentlessly progressive and disabling disorder.

## 8. Therapeutic Approaches

Based on the presence of inflammatory infiltrates in skeletal muscle and the occurrence of circulating anti-cN-1A antibodies, a lot of efforts have been made to develop potential therapies aiming at modulating the inflammatory responses in sIBM. Immunosuppressive and immunomodulatory drugs targeting different metabolic pathways or molecular players fundamental for immune cells or specifically required for cytotoxic T cell effector functions have been evaluated (Table 2). These include prednisone, azathioprine, methotrexate, IVIG, anti-T lymphocyte globulins (ATG), interferon beta 1a, etanercept, infliximab, alemtuzumab, anakinra, natalizumab, rituximab, and canakimumab [10,219,220,221,222,223,224,225,226,227,228,229,230,231,232,233,234,235,236]. These agents have been used alone or in different combinations in several observational studies and clinical trials. However, so far, targeting immune responses has demonstrated only minimal or absent beneficial effects on patients. Some reports even suggest that prednisone and other immunosuppressant drugs may instead have negative effects on disease progression [10,220,221]. Despite these discouraging and concerning results, prednisone is still the most prescribed for sIBM. Immunoglobulins (IVIG) were found to stabilize dysphagia, so they are also used in clinical practice, despite studies that have not been able to conclude their efficacy in improving limb muscle weakness [223,224,225,237,238]. Other immunomodulatory drugs, such as Sirolimus (rapamycin) and ABC008, are still currently under investigation in clinical trials (Table 2).

The degenerative features of sIBM provided the rationale for designing potential therapies targeting non-inflammatory pathways, such as proteostasis, with the goal of decreasing protein aggregation and enhancing muscle mass. Lithium has been studied because of its ability to induce autophagy and inhibit glycogen synthase kinase-3 (GSK), which is involved in tau and APP phosphorylation [65]. Sirolimus, which is still under investigation, can not only block the activity of effector T cells but also induce autophagy [239]. Arimoclomol has been tested based on its ability to increase heat shock response and decrease protein aggregates in in vitro models of sIBM [240]. Efforts have been made to promote muscle growth by inhibiting the myostatin pathway with a monoclonal antibody against the myostatin receptor (Bimagrumab) or increasing protein synthesis with anabolic androgenic steroids (oxandrolone) [241,242,243].

Other therapeutic strategies have been considered, including gene therapy and, more recently, stem cell therapy. Delivery of follistatin gene, encoding a glycoprotein that inhibits the binding of myostatin to ActRIIB receptors on muscle cells, in quadriceps muscle using adeno-associated virus (AAV) has shown promising but still not conclusive results [244,245,246]. The safety and efficacy of adipose-derived regenerative cell injections in the forearm and thigh for the treatment of sIBM are currently under investigation (Table 2) [247].

Besides pharmacological agents, different exercise regimens have been assessed as possible therapeutic approaches for sIBM [248,249,250,251,252,253]. These studies eliminated the concern of exercise being potentially harmful and demonstrated that physical activity ameliorates strength in sIBM patients. Still, exercise programs tested in these studies did not improve mobility in the patients.

## 9. Future Perspective in sIBM Research and Therapy

Despite many efforts, to date, there are no effective treatments for sIBM, and there is an urgent need for disease-modifying therapies for patients. Interestingly, several of the described drugs had shown promising effects in preclinical studies, but only some of them demonstrated slightly positive but still not significant effects when evaluated on patients. The poor translation of preclinical findings into clinical practice could be due to several reasons. The lack of appropriate pre-clinical models of sIBM certainly represents a major obstacle, not only to successfully develop effective therapies but also to understand disease mechanisms [254]. Some of the drugs that have been tested in clinical trials are based on data obtained on valosin-containing protein (VCP) mutant mice model of IBM associated with Paget’s disease of the bone, fronto-temporal dementia, and amyotrophic lateral sclerosis (IBMPFD/ALS) [240,255,256]. Unfortunately, these mice reproduce only partially sIBM pathology. Therefore, efforts should be made to develop models that better recapitulate the disease, and that can be more reliable models to be used in preclinical studies [214,257]. Also, the rarity of sIBM, the often late diagnosis of the disease, and the clinical heterogeneity between patients represent major challenges in the design of clinical trials.

Increased understanding of the pathogenesis, specifically the causative relationship and the crosstalk between inflammatory and degenerative mechanisms, as well as exposing novel molecular players in the disease, could strongly benefit the design of future therapies for sIBM. Considering the lack of appropriate pre-clinical models, alternative technologies need to be exploited. Omics approaches have been recently contributing to improving our understanding of the disease [67,258,259,260,261,262,263,264]. Proteomics analysis performed on skeletal muscle biopsies of patients with sIBM has revealed alterations of pathways related to oxidative stress and regulation of apoptosis [261]. Selective analysis of rimmed vacuole protein composition led to the identification of FYCO1, an adaptor protein that forms a complex with LC3 and Rab7 and regulates the transport of macroautophagic/autophagic vesicles [260,265]. Interestingly, rare missense variants in FYCO1 were detected at higher frequency in sIBM patients, further supporting alterations in the endolysosomal pathway that take part in the disease [260]. Transcriptomics studies have also been carried out on skeletal muscle tissue from patients with sIBM, leading to the identification of alterations in factors involved in RNA splicing, such as hnRNPA1, hnRNPA2/B1, hnRNPC1/C2, hnRNPH [67]. These proteins, which are known to interact and be required for TDP43 splicing activity, were also found mislocalized in the cytoplasm of sIBM muscle fibers. In agreement with this finding, changes in tau transcript splicing have been detected in this patient cohort, suggesting that alterations in RNA metabolism may have a role in the disease [67]. Metabolomic and transcriptomic analyses have been used in combination to uncover alterations in histamine biosynthesis, certain glycosaminoglycan biosynthesis, carnitine, and creatine metabolism, providing the rationale for the identification of mast cells in sIBM muscle biopsy [259]. A more systemic approach has been adopted in a recent study where metabolomic and transcriptomic analyses were performed on a set of tissues, cells, and biological fluids obtained from patients with sIBM [258]. This strategy uncovered systemic metabolic alterations occurring in sIBM and identified L-pyroglutamic and orotic acids in urine as a highly specific and selective biomarker signature for sIBM [258]. Single-cell RNA sequencing (scRNAseq) has been recently applied to characterize the muscle-infiltrating T cells and peripheral blood memory T cells in patients with IIM, including subjects with sIBM [266]. This study confirmed previous observations, including the presence of tissue-resident memory T-cells and of clonally expanded T cells in muscle biopsies of IIM patients. Even though only a few patients were analyzed, and transcriptomic signatures of different IIM need to be confirmed in a higher number of patients, the study showed the feasibility and power of scRNAseq to investigate IIM [266]. The role of miRNA in sIBM has been recently exposed [267,268]. Analysis of miRNA expression profile in the skeletal muscle of patients with IIM using NanoString led to the identification of a specific miRNA signature in sIBM [267]. miR-150-5p, a miRNA that regulates B- and T-cell differentiation, was found specifically increased in sIBM compared to healthy subjects and patients with other IIM and proposed as a possible biomarker for sIBM. This finding agrees with the presence of highly differentiated T and B cells in sIBM muscles. Also, miRNA involved in muscle regeneration have been found to be increased in sIBM [267]. Machine learning techniques are also emerging as powerful tools to improve our understanding of the disease, discover new biomarkers of sIBM, and expose new potential therapeutic targets [269]. Exploring other biological pathways, besides immune responses and proteostasis, that are altered in sIBM could help in designing novel potential treatments. In this regard, mitochondrial alterations have been gaining attention as an important feature of sIBM pathology [97,270]. Interestingly, metabolomics studies in blood revealed common metabolic changes in sIBM and primary mitochondrial myopathies, indicating the potential for shared treatment strategies [103]. These findings suggest that interventions promoting mitochondrial functions and reducing the effects of mitochondrial dysfunction might be beneficial for sIBM patients [97]. On this line, the mitochondria-homing drug mitochonic acid-5 (MA-5), which augments cellular ATP level and decreases mitochondrial ROS (mtROS) generation, was recently shown to improve morphological alterations, dynamics and functions in primary myoblasts obtained from the muscle of sIBM patients, offering interesting clues toward a possible alternative strategy for treating sIBM [100]. Recently, the transfer of healthy mitochondria has been proposed as a novel possible therapeutic strategy to treat diseases associated with mitochondrial defects [271]. A clinical trial in Korea is currently evaluating the effect of allogeneic mitochondria transplantation in patients with dermatomyositis, which is also characterized by mitochondrial defects, even though in less extent compared to sIBM [272]. Even though many technical and ethical aspects still need to be sorted out, in the future, mitochondrial replacement therapy (MRT), also called mitochondrial transplantation, may become a therapeutic avenue for patients affected by sIBM.

sIBM is a complex, multifaceted disease in which several biological components come together in an intricate net of interactions. Cutting-edge technologies like omics and single-cell techniques are certainly helping to unveil novel players and pathways involved in disease pathogenesis. Still, these powerful techniques per se might not clarify the causal relationships between these different components of the disease unless used within specific experimental designs.

## 10. Conclusions

sIBM is a multifactorial disorder characterized by the coexistence of inflammation and degeneration. The pathological features of the disease have been extensively characterized, and alterations in specific cellular pathways have been proposed as contributors to disease pathogenesis. Notably, many of the cellular and molecular processes that are altered in sIBM are also affected by aging, which likely has an influence on disease onset and progression.

Despite the fact that some cellular and molecular mechanisms have been uncovered, the etiology of the disease is still unknown, and the causal relationship between autoimmune and degenerative responses has not yet been established. Further studies are needed to illuminate these aspects with the long-term goal of designing effective therapies for this progressive and debilitating disease.

## Figures and Tables

**Table 1:** Pathways and molecular players involved in sIBM.

Process	Molecular Players and Their Alterations in sIBM
Degenerative Features	
Protein aggregation in muscle fibers	proteins prone to unfold/misfold (Aβ protein, tau, α-synuclein, prion, presenilin, etc.) proteins involved in AβPP processing (BACE1 and BACE2 and the γ-secretase complex) proteins involved in tau phosphorylation (ERK, CDK5, GSK-3β, and casein kinase 1) proteins involved in endoplasmic reticulum stress (GRP78 and GRP94) proteins participating in oxidative/nitrative stress (nitrotyrosine, NOS, and SOD1) regulators of proteostasis (heat shock proteins, 26S proteasome components, ubiquitin, p62/ SQSTM1, and ubiquitin) proteins involved in RNA metabolism (RNA polymerase II, FUS, TDP-43, VCP, c-Jun, NFkB hnRNPA1, hnRNPA2/B1, hnRNPC1/C2, and hnRNPH)
Impairment of ubiquitin–proteasome system (UPS) and autophagy	HSP70 and αB-crystallin increased expression of 26S proteasome subunits decreased activity of proteasomal proteolytic enzymes aggresome formation Atg8/LC3 positive autophagic vacuoles containing AβPP and Aβ increased mRNA or protein expression of the lysosomal proteins M6PR, clathrin, hApg5, and hApg12 increased mRNA and protein expression of p62/SQSTM1 decrease activity of the lysosomal enzymes cathepsin D and B accumulation of LC3II and p62 increased of NRB1, Beclin 1, ATG5, and clathrin upregulation of components of the chaperone-mediated autophagy (Hsc70 and LAMP2A)
Endoplasmic reticulum (ER) stress and Unfolded Protein Response (UPR)	increased expression of calnexin, calreticulin, BiP/GRP78, GRP94, ERp72, ATF4, and CHOP alternative splicing of XBP1 N-terminal fragment of activating transcription factor 6 (ATF6) upregulation of homocysteine-induced ER protein (Herp) increased expression of myostatin precursor protein (MstPP)
Mitochondrial abnormalities	mitochondria proliferation COX-negative fibers and RRFs mtDNA alterations impaired oxidative phosphorylation (OXPHOS) reduced oxygen consumption rate/increased extracellular acidification rate reduction in the protein levels of complex I, III, complex IV alteration of mitofusins (Mfn) and optic atrophy 1 (OPA1) (mitochondria morphology) changes miR-133 and PGC1α (mitochondrial biogenesis)
Oxidative stress	accumulation of nitrotyrosine, nNOS, iNOS, MnSOD, and CuZnSOD in vacuolated fibers increased mRNA and protein levels of insulin-like growth factor I (IGF-I) and DJ-1 ROS/RNS
Nuclear degeneration	emerin and lamin A/C detected within rimmed vacuoles accumulation of TDP-43 and hnRNPs in the cytoplasm
Aged skeletal muscle milieu	impaired proteostasis and autophagy changes in nuclear architecture altered expression of the myogenic regulatory factors MyoD and myogenin mtDNA mutation senescent fibro-adipogenic progenitors (FAPs)
Inflammatory Features	
T cells	CD8^+^ T cells express perforin, granulysin, granzyme B, and natural killer markers CD16 or CD56 increased of CXCL-9, CXCL-10, IL-12, CCL-2, and IL-1RA and IFN-γ in situ clonal expansion highly/terminally differentiated and exhausted T cells (expression of CD224 and CD57) exhausted T-cells with increased expression of inhibitory receptors PD1, LAG-3, TIM-3, TIGIT Tregs reduction in blood and skeletal muscle
Plasma cells and antibody-mediated immune response	increased expression of CD138^+^ plasma cells in situ oligoclonal expansion, isotype switching, and accumulated somatic mutations increased serum levels of B-cell activating factor antibodies against cN1A
Macrophages and dendritic cells	increased expression of CD74, CD163, and STAT1 in macrophages increased interferon-induced protein ISG15 and IRF8 on MHCII^+^ macrophages muscle infiltration with myeloid DC (mDCs) that co-localize with T cells
Aging of the immune system and sIBM	accumulation of terminally differentiated T cells restricted T cell receptor (TCR) repertoire Tregs peripheral deficiency

**Table 2:** Treatment for sIBM that are currently used in the clinical practice or that have been previously studied or are still under study in clinical trials.

Agent	Description
Immunosuppressive	
Prednisolone	Glucocorticoid binds to glucocorticoid receptor, inhibits pro-inflammatory signals, decreases leukocyte migration to the site of inflammation, promotes anti-inflammatory effects
Azathioprine	Inhibits purine synthesis with consequent decrease in DNA, RNA, and protein synthesis. Inhibits CD28 signals in T and B cells
Methotrexate	Inhibition enzymes are responsible for nucleotide syntheses such as dihydrofolate reductase, thymidylate synthase, aminoimidazole caboxamide ribonucleotide transformylase (AICART), and amido phosphoribosyltransferase. Prevents cell division
IVIg	Pooled of polyclonal IgG from healthy donors. Bind pathogenic autoantibodies or cross-react with various antigenic peptides
Anti-T lymphocyte globulin (ATGAM)	Purified rabbit anti-human thymocyte antibodies. Blocks T cell-mediated immune reactions
β-Interferon 1a	Cytokine. Immunomodulation function. Promote anti-inflammatory immune response
Etanercept	TNF receptor fusion protein with Fc portion of human IgG. Binds to and inhibits TNFα-mediated immune responses
Infliximab	TNFα-inhibiting monoclonal-antibody. Block TNFα-mediated immune responses
Alemtuzumab	Anti-CD52 monoclonal antibody. Depletes circulating T and B lymphocytes
Rituximab	Anti-CD20 monoclonal antibody. B cell-depletion
Anakinra	IL1 receptor antagonist blocks IL1β binding to its receptors
Sirolimus	Mammalian target of rapamycin (mTOR) inhibition. Promotes autophagy. Blocks effector T cells
ABC008	Anti-KLRG1 antibody. Depletion of highly cytotoxic T cells, without affecting regulatory and central memory T cells
Simvastatin	Statin, HMG-CoA reductase inhibitor. Cholesterol-lowering drug with multiple other actions, including inhibition/modulation of inflammatory responses, enhancement of endothelial function, modulation of progenitor cells, antioxidant and neuroprotective effects
Non-Immunosuppressive	
Lithium	Glycogen synthase kinase-3 (GSK) inhibition. Modulates proteostasis and immune responses
Oxandrolone	Synthetic anabolic androgen steroid. Skeletal muscle mass enhancer
Follistatin gene therapy	Natural inhibitor of the myostatin receptor. Skeletal muscle mass enhancer
Bimagrumab	Monoclonal antibody. Binds competitively to activin type II receptors (ActRII, the myostatin receptor). Skeletal muscle mass enhancer
Arimoclomol	Heat shock protein inducer. Promotes protein folding
Adipose-Derived Regenerative Cells	Autologous adipose tissue-derived stem cells
